# From Intense Rejection to Advocacy: How Muslim Clerics Were Engaged in a Polio Eradication Initiative in Northern Nigeria

**DOI:** 10.1371/journal.pmed.1001687

**Published:** 2014-08-05

**Authors:** Sani-Gwarzo Nasir, Gambo Aliyu, Inuwa Ya'u, Muktar Gadanya, Muktar Mohammad, Mahmud Zubair, Samer S. El-Kamary

**Affiliations:** 1 Federal Ministry of Health, Abuja, Nigeria; 2 Health and Human Services, Federal Capital Territory, Abuja, Nigeria; 3 National Primary Health Care Development Agency, Abuja, Nigeria; 4 Aminu Kano Teaching Hospital, Kano, Nigeria; 5 Department of Epidemiology and Public Health, University of Maryland School of Medicine, Baltimore, Maryland, United States of America

## Abstract

Gambo Aliyu and colleagues describe an approach to eradicating polio in Northern Nigeria by engaging Muslim clerics in influencing community perceptions.

*Please see later in the article for the Editors' Summary*

**Summary Points**

- Of the several setbacks suffered by the polio eradication initiative in Nigeria, vaccination rejection by Muslim clerics (imams) is perhaps the most profound.
- Anti-polio propaganda, misconceptions, and violence against vaccinators at the community level present huge challenges to polio eradication in Nigeria and globally.
- However, the intense opposition to polio vaccination is systematically being reversed by the active engagement of imams to promote uptake of polio vaccination in areas worst hit by the disease.
- A coalition campaign involving imams, Islamic school teachers, traditional rulers, doctors, journalists, and polio survivors is gradually turning the tide against polio vaccine rejection in northern Nigeria.
- Innovative engagement and the coalition campaign at the community level should be part of the focus of the polio eradication initiative in Nigeria.

## Background

Before the allegations of polio vaccine contamination a decade ago, Nigeria's polio eradication initiative (PEI) was affected by traditional barriers such as inadequate funds, poor coverage, poor supervision, broken cold chains, and lack of community mobilization and ownership. However, after the allegations of contamination of the vaccine with HIV, carcinogens, and sterilizing agents, the program was completely halted in some states [1]–[3], leading to the reversal of gains made in the global PEI. Following this program interruption, infection reemerged in 20 countries in which the polio virus had been previously eradicated [4]–[6].

Subsequently, religious, safety, and fertility concerns became the main barriers to polio vaccination in most of northern Nigeria, one of the few remaining holdouts of the disease worldwide [2],[3],[7]. Even in areas with better uptake, failure to engage parents and discuss why a fully vaccinated child may develop polio disease, for instance, increased parents' negative perception of the program [8]. Rebuilding community trust, understanding, cooperation, and support—all of which were lost to the groundless controversy on the vaccine's safety—became necessary.

In 2008, a community communication and awareness enhancement methodology known as Majigi (a roadside film show) was pilot-tested in Gezawa, Kano State, located in northwestern Nigeria [9]. In this pilot campaign, coordinated by the National Primary Health Care Development Agency (the agency responsible for developing primary health care programs and policies in Nigeria), the participation of imams (Muslim clerics) was solicited through the traditional rulers to mobilize people at the community level [9]. Active participation of the traditional rulers in PEI in northern Nigeria became a reality after the office of His Royal Highness, the Sultan of Sokoto, supported this program. The sultan is the current Muslim spiritual leader in Nigeria, with a strong influence on the dominant Muslim community of the region. The sultan title is of historical significance and originates from the time when sultans were the leaders of the Islamic caliphate that ruled northern Nigeria in the precolonial period. The sultan's support was a major milestone, given that his opinion carries tremendous weight with Fulani and Hausa people from northern Nigeria, and was pivotal to the success of the initiative in this region.

The encouraging outcomes of the Majigi polio mobilization campaign led to the formal launch of the Northern Traditional Leaders Committee for Primary Health Care and Polio Eradication in 2009 by the sultan, which set the stage for the engagement of the imams. This article discusses the involvement and impact of the imams in promoting polio vaccination and the emerging coalition of polio vaccine promoters at the community level in northern Nigeria.

## Engagement of Imams in the Polio Eradication Initiative

**Figure 1:**
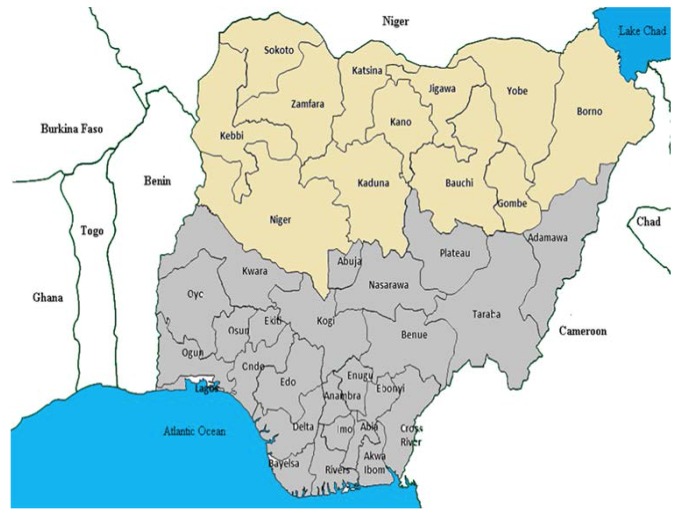
Poliomyelitis-prevalent states of northern Nigeria. A line map of Nigeria showing the states (gold) where incident poliomyelitis cases were detected.

In 2010, the National Primary Health Care Development Agency constituted a team of experts, consisting of mainly Muslim scientists, to work with the traditional leaders committee under the leadership of the Sultan to reach out to the various Muslim group leaderships. This team was named the National Facilitation Team, and four of the authors were members of this team, headed by the first author. The team engaged, educated, and solicited for the support of imams and their community followers for the PEI. The team began with mapping out the high-risk local government areas (LGAs) within the 12 polio-prevalent states of northern Nigeria, namely, Bauchi, Borno, Gombe, Jigawa, Yobe, Kaduna, Kano, Katsina, Kebbi, Niger, Sokoto, and Zamfara (Figure 1).

A total of 85 high-risk LGAs were selected based on the presence of polio cases, low vaccination coverage, poor compliance, and location next to LGAs with polio cases. In collaboration with the traditional leaders committee, the facilitation team established the Northern Islamic Religious Leaders Contact Group and organized training sessions for selected imams from different communities in the LGAs with high polio risk. This contact group of prominent imams from various Islamic sects played the critical role of recruiting fellow imams from the high-risk LGAs. Not all the imams contacted by the religious leaders contact group agreed to attend the training sessions. There were a few skeptics and vocal outright rejectionists, for whom the assistance of local traditional leaders was sought to approach them and invite them to attend the training program. Some imams came to the sessions on their own out of curiosity, while others came to prove us wrong because of the confidence they had in their views.

The selection of imams was reflective of the population size and distribution of mosques in the high-risk communities. The training focused on Jumu'a imams, who give the weekly Friday sermon and lead the prayer on that day. They are traditionally the most knowledgeable religious scholars and the most influential and respected in the community, compared to the regular prayer imams, who lead only the regular prayer five times a day and do not give religious sermons.

The participants were serially trained in weekly sessions for ten weeks. Materials for the training were jointly developed by the facilitation team and the religious leaders contact group. The materials included Islamic rulings (fatwas) on the concept of vaccination and 13 well-written Friday sermons in Arabic and native languages on disease prevention, health, and related matters for the imams to adopt, edit, or rewrite to suit their audiences. On the topic of poliomyelitis, the facilitation team prepared Majigi sessions preceded by PowerPoint presentations and a computer simulation model of the polio virus and its routes of transmission, early signs, symptoms, and complications. Films were shown of recorded interviews and emotional movies of survivors of the disease sharing their experiences and frustrations of living with deformities from the disease. There were also recorded video interviews of the survivors' care providers and important advice to the community and parents on polio vaccination.

**Figure 2:**
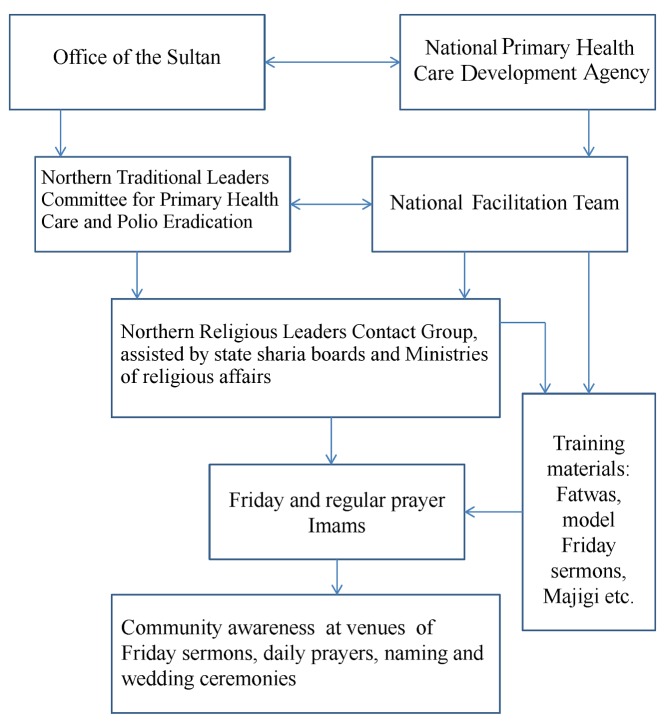
Flow chart for the engagement of imams in the poliomyelitis eradication initiative. A flow chart illustrating the different groups that facilitated the recruitment of the Jumu'a (Friday) and regular prayer imams into the PEI. Double-direction arrows indicate collaboration or consultation, while single-direction arrows denote selection or application.

At the end of the training sessions, the majority of the imams were highly receptive to the idea of being engaged in the PEI, with sincere appreciation, a sense of urgency, or even disappointment about the delay in engaging them. Each Jumu'a imam was requested to engage and share information with at least five regular prayer imams within his community. Together, the imams were expected to deliver the message repeatedly after daily prayers, and at public gatherings such as naming and wedding ceremonies. The imam engagement process is summarized in Figure 2.

## Community Campaign for Polio Eradication

Local efforts involving different pressure groups are currently being used to get children from very high-risk and resistant wards (districts) vaccinated in northern Nigeria. In addition to the Muslim clerics, the campaign engages the services of polio survivors, medical doctors, Quranic/Islamic school teachers, Christian clerics (for vaccine-refusing Christian communities), traditional rulers, and street entertainers (drummers). From June to July 2013, over 3,256 refusing households from high-risk wards in Sokoto State were visited by members of the coalition campaign team, with more than 7,825 children vaccinated [10]. Intensified campaigns at the community level are yielding favorable outcomes; for the first time some states in the north are now polio-free, while some are said to be considering enacting laws to promote compliance with routine polio vaccinations.

## Changes in the Number of Polio Cases in Nigeria from 2009 to 2013

According to the acute flaccid paralysis (AFP) surveillance data for polio from the National Primary Health Care Development Agency, there were 384 new wild poliovirus (WPV) cases reported in Nigeria in 2009, and of those, 322 (84%) occurred in the 12 polio-prevalent states of the north. The number of reported cases plummeted in 2010 to 21 from 384 (a 95% decline) nationwide, and to 19 from 322 (a 94% decline) in the polio-prevalent states. However, in 2011 this trend was not sustained, and there was an increase to 62 new WPV cases in eight polio-prevalent states. The numbers continued to rise in 2012, with 122 WPV cases, of which 118 were reported in ten polio-prevalent states. There were, however, signs of a decline again in 2013, when, by the end of October, only 49 cases were reported nationwide, with 46 in five polio-prevalent states.

**Figure 3:**
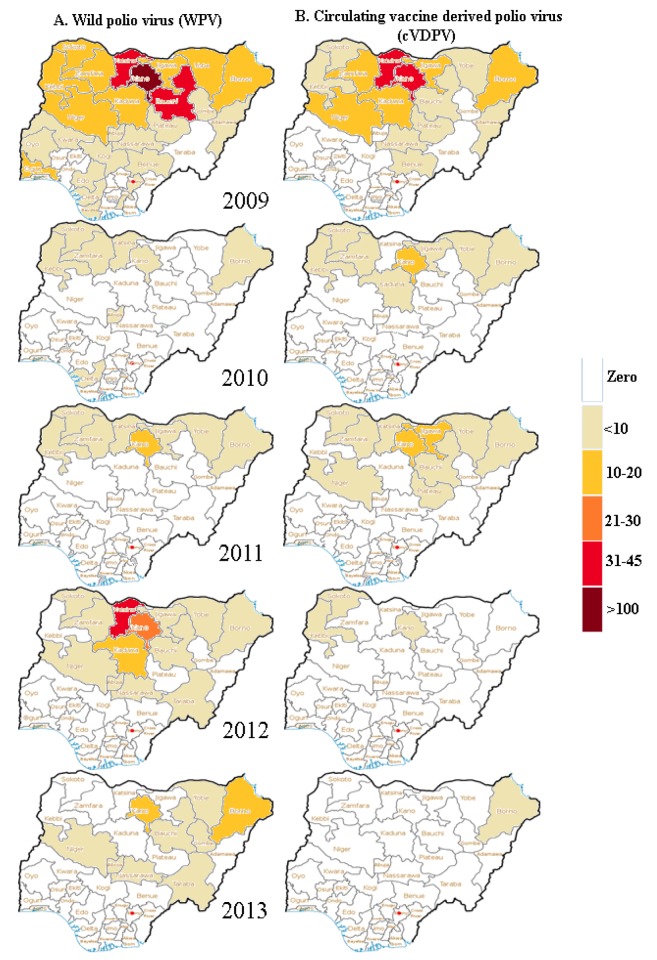
Pattern of occurrence of cases of wild poliovirus and circulating vaccine-derived poliovirus in Nigeria from 2009 to 2013. This figure summarizes the number of incident (A) WPV and (B) cVDPV cases in Nigeria during the period from 2009 to 2013. The color scheme represents the increasing intensity of incidence from white (zero) to light yellow, to gold, to orange, to red, and to dark red.

From the same AFP surveillance data, the prevalence of circulating vaccine-derived poliovirus (cVDPV) followed a similar pattern. A total of 154 cases were reported across the country in 2009, 151 from 11 of the 12 polio high-risk states. The number of cases decreased to 27 in 2010 but rose to 35 in 2011, then decreased to eight in 2012, and only one case was reported in 2013. Figure 3 summarizes the number of new WPV and cVDPV cases in Nigeria during the period from 2009 to 2013.

## Impact of Engaging Imams and the Way Forward

The engagement of the traditional and religious leaders in the polio immunization campaign in northern Nigeria was vital to the success of the country's PEI and primary health care programs. The recent extension of the campaign to include polio survivors, Quranic school teachers, and entertainers, among others, is providing access to families that are hard to reach and non-compliant. Following the introduction of the campaign at the community level, occurrence of all types of new polio cases drastically dropped to the lowest level. Although the separate impact of the government's renewed political commitment at all levels may be hard to quantify, the influence of religious leaders in northern Nigeria was enormous. In the northern Nigerian Muslim communities, radio and television jingles, and even the roadside film show used in the Majigi campaign to educate the community, were not as powerful as Friday sermons and after prayer talks in mobilizing the community. Approaching the religious leaders through a group they trusted and respected (traditional rulers) appeared to be a missing link in the campaign for polio eradication in Nigeria.

The decline in the occurrence of polio cases was, however, not consistent over the five-year period. The number of cases of WPV increased in 2012, while the three polio-prevalent states of Bauchi, Kaduna, and Niger that did not report any cases of WPV in 2010 and 2011 reported new cases of WPV in 2012 (Figure 3). Among the 12 polio-prevalent states, Katsina recorded the highest number of WPV cases in 2012, followed by the most populous state, Kano. The bump in the occurrence of WPV in 2012 may be linked to the single attack on and killings of polio vaccinators in Kano. This attack was widely believed to be carried out by members of an Islamic insurgent group, and temporarily dampened the drive for house-to-house vaccination in most of the polio-prevalent states [11]. Also, refusing wards or households in some of the high-risk LGAs, despite the engagement of the religious leaders, could have served as sources of fresh outbreaks; these households may represent die-hard rejectionists whom imams were unable to convince, or they may belong to a different religious sect than the imam, or they may have witnessed a case or cases of vaccine-induced poliomyelitis.

The occurrence of new cases infected with the cVDPV strain declined consistently over the five-year period, indicating improved acceptance and uptake of polio vaccinations, with the population as a whole better immunized and less susceptible to poliovirus [12],[13]. However, the risk of occurrence of infection with this strain remains as long as live attenuated vaccine is used for polio vaccination [14],[15]. Despite the high number of WPV cases reported from Katsina in 2012, no case of cVDPV was reported from the state in 2010 and 2012, and by the end of 2013, our investigation shows that this high-risk state did not report any new cases of either WPV or cVDPV.

The prevailing religious conflict in the northeastern region of the country in the polio-prevalent states of Borno and Yobe, coupled with the history of prior attack on vaccinators, could undermine the current momentum for polio eradication across northern Nigeria. Failure of vaccinators to make household visits in this region due to safety concerns may lead to explosive outbreaks similar to those seen recently in Somalia and Syria [16],[17]. In addition to the door-to-door effort, parents should be encouraged to take their children to the local health facilities for routine immunizations including vaccination against polio. Given the evidence from northern Nigeria that access to oral polio vaccine through the World Health Organization's Expanded Programme for Immunization improves coverage [18], it is worth considering ways to incentivize parents to promote patronage for the routine immunization program, such as supporting their transportation to the facilities in the most affected locations. Reducing publicity around the program at the community level may also reduce the risk of attacks on vaccinators, and promoting bottom-up (rather than top-down) engagement, where the community sees polio as a social problem for which it has direct responsibility, could improve acceptance of the program [19].

The expansion of polio immunization advocacy to include Quranic school teachers, doctors, polio survivors, and entertainers should be extended to groups such as village health committees, which can help drive demand, not only for polio vaccines, but also for other priority services. Expanding activities and concerns beyond polio may help to dispel some of the suspicions around why polio has become so important, particularly in areas where strong anti-polio sentiments persist. This multifaceted approach is needed for parents who remain suspicious despite the intervention of the religious and traditional leaders, and for late adopters of behavior change, for whom special home visits and new approaches may be necessary. While these individuals may constitute a negligible percentage of the population, they are crucial in the effort to eradicate polio completely.

A different approach may, however, be required for the nomads who constantly move back and forth from north to south in search of pasture. This lifestyle is believed to increase nomads' vulnerability to polio, and these groups could serve as reservoirs of infection for the reemergence of the disease even after transmission is successfully interrupted [20], although recent evidence has shown that nomadic lifestyle is not significantly associated with a failure to vaccinate [18]. Engaging the leadership of the Miyetti Allah Cattle Breeders Association of Nigeria to create demand for routine health services in various nomad communities may improve polio vaccination coverage in this population.

In conclusion, this campaign demonstrates that traditional leaders in Nigeria could be relied upon to mobilize religious clerics, who in turn educated and mobilized the community. Expanding the focus of the existing coalition campaign, which encourages parents to accept the vaccine initiative and have their children immunized, to creating awareness in the community to demand polio vaccination will have a tremendous impact on the PEI in Nigeria, and the sustainability of such efforts remains the greatest challenge to eradication of polio from Nigeria.

## Abbreviations

AFP: acute flaccid paralysis
cVDPV: circulating vaccine-derived poliovirus
LGA: local government area
PEI: polio eradication initiative
WPV: wild poliovirus
